# Qingda Granule Attenuates Angiotensin II-Induced Blood Pressure and Inhibits Ca^2+^/ERK Signaling Pathway

**DOI:** 10.3389/fphar.2021.688877

**Published:** 2021-07-29

**Authors:** Meizhu Wu, Xiangyan Wu, Ying Cheng, Zhiqing Shen, Xiaoping Chen, Qiurong Xie, Jianfeng Chu, Jiapeng Li, Liya Liu, Lihui Wei, Linzi Long, Qiaoyan Cai, Jun Peng, Aling Shen

**Affiliations:** ^1^ Academy of Integrative Medicine, Fuzhou, China; ^2^ Chen Keji Academic Thought Inheritance Studio, Fuzhou, China; ^3^ Fujian Key Laboratory of Integrative Medicine on Geriatrics, Fujian University of Traditional Chinese Medicine, Fuzhou, China; ^4^ Department of Physical Education, Fujian University of Traditional Chinese Medicine, Fuzhou, China; ^5^ Department of Geriatrics, Xiyuan Hospital, China Academy of Chinese Medical Sciences, Beijing, China

**Keywords:** Qingda granule, angiotensin II, hypertension, vascular smooth muscle cells, Ca^2+^/ERK signaling pathway

## Abstract

**Objective:** As a well-known traditional Chinese medicine formula prescribed by academician Ke-ji Chen, Qingda granule (QDG) lowered the blood pressure of spontaneously hypertensive rats and attenuated hypertensive cardiac remodeling and inflammation. However, its functional role and underlying mechanisms on hypertensive vascular function remain largely unclear. This study aims to assess the effects of QDG treatment on Angiotensin II- (AngII-) induced hypertension and vascular function and explore its underlying mechanisms both *in vitro* and *in vivo*.

**Methods:** In an *in vivo* study, 25 male C57BL/6 mice were randomly divided into five groups, including Control, AngII, AngII + QDG-L, AngII + QDG-M, and AngII + QDG-H groups (*n* = 5 for each group). Mice in AngII and AngII + QDG-L/-M/-H groups were infused with AngII (500 ng/kg/min), while in the Control group, they were infused with saline. Mice in AngII + QDG were intragastrically given different concentrations of QDG (0.5725, 1.145, or 2.29 g/kg/day), while in Control and AngII groups, they were intragastrically given equal volumes of double distilled water for 2 weeks. Blood pressure was determined at 0, 1, and 2 weeks of treatment. Ultrasound was used to detect the pulse wave velocity (PWV) and HE staining to detect the pathological change of the abdominal aorta. RNA sequencing (RNA-seq) was performed to identify the differentially expressed transcripts (DETs) and related signaling pathways. IHC was used to detect the expression of p-ERK in the abdominal aorta. Primary isolated rat vascular smooth muscle cells (VSMCs) were used to assess the cellular Ca^2+^ release and activation of the ERK pathway by confocal microscope and western blotting analysis, respectively.

**Results:** QDG treatment significantly alleviated the elevated blood pressure, the PWV, and the thickness of the abdominal aorta in AngII-induced hypertensive mice. RNA-seq and KEGG analyses identified 1,505 DETs and multiple enriched pathways (including vascular contraction and calcium signaling pathway) after QDG treatment. Furthermore, confocal microscope showed that QDG treatment partially attenuated the increase of Ca^2+^ release with the stimulation of AngII in cultured VSMCs. In addition, IHC and western blotting indicated that QDG treatment also partially alleviated the increase of phospho-ERK levels in abdominal aorta tissues of mice and cultured VSMCs after the infusion or stimulation of AngII.

**Conclusion:** QDG treatment attenuated the elevation of blood pressure, abdominal aorta dysfunction, pathological changes, Ca^2+^ release, and activation of the ERK signaling pathway.

## Introduction

Hypertension is the most important modifiable risk factor for all-cause morbidity and mortality worldwide and associated with an increased risk of cardiovascular diseases such as coronary artery disease, stroke, heart failure, and chronic kidney disease, which are the leading causes of death in China (Wenzel et al., 2011; Wang et al., 2018; Benjamin et al., 2019). The main damage of hypertension is that the long-term pressure overload causes the functional and structural change of the target organs, including the aorta, heart, and kidney, which ultimately causes organ failure (Touyz 2005; Rubattu et al., 2019; Di Palo and Barone, 2020). Despite the improvement of awareness and treat (Collaborators 2017), exploring more effective pharmacological therapies to control blood pressure and alleviate organ damage is an urgent issue in this area.

The etiology of hypertension involves a complex interplay of various elements, including genetic, neurohumoral, and environmental factors, among which the renin-angiotensin-aldosterone system (RAAS) plays a critical role in the pathogenesis of hypertension (Elliott 2007; Fujii et al., 2007; Oparil et al., 2018). The RAAS has wide-ranging effects on blood pressure regulation, mediating vasoconstriction, endothelial dysfunction, and vascular injury through its most pharmacological factor angiotensin II (AngII) binding with its receptor, that is, AngII type I receptor (AT1R) (Gasc et al., 1994; Garg and Yusuf, 1995; Touyz 2005; Montezano et al., 2014). Vascular dysfunction is a crucial pathological process during the development of hypertension, which in turn promotes its progression (Pan et al., 2018; Lang et al., 2020; Lu et al., 2020). Once AngII binds to the extracellular part of AT1R on the vascular smooth cell membrane, its actives the “classical” G protein-dependent signaling pathway, which triggers vasoconstriction, in which calcium efflux from the sarcoplasmic reticulum and downstream ERK signaling pathway play an important role (Sugden and Clerk, 1997; Seko et al., 2003; Yan et al., 2003; Mehta and Griendling, 2007; Wilson et al., 2015). Therefore, rescuing the vascular functional and structural change at the early stage of hypertension may be an important means to interfere with the progression of hypertension.

Qingda granule (QDG) is a simplified formulation based on Qingxuan Jiangya Decoction (QXJYD), prescribed by academician Ke-ji Chen, which is a well-known traditional Chinese medicine formula to treat hypertension for more than 60 years in China (Yu et al., 2020; Zhang et al., 2020; Cheng et al., 2021). QDG is composed of *Gastrodia elata* Blume (Tianma), *Uncaria rhynchophylla* (Miz.) Miz. ex Havil. (Gouteng), *Scutellaria baicalensis* Georgi (Huangqin), and *Nelumbo nucifera* Gaertn (Lianzixin) in a ratio of 12: 10: 6: 5. Many active components in QDG derived from a famous traditional Chinese medicine for hypertension therapy, such as baicalin, gastrodin, and uncarine, have the anti-hypertension effect (Dai et al., 2017; Qian et al., 2020). Our previous studies have shown that QDG significantly attenuated the elevation of blood pressure by inhibiting vasoconstriction and promoting vasorelaxation in spontaneously hypertensive rats (SHRs) (Huang et al., 2019). We also have shown that QDG attenuated elevated hypertension and inhibited the proliferation of vascular smooth muscle cells (VSMCs) by suppressing MAPK signaling pathway in AngII-treated mice (Yu et al., 2020). In addition, QDG alleviated the hypertensive cardiac inflammatory infiltrates and cardiac hypertrophy and remodeling in hypertensive rats or mice model (Wu et al., 2020; Yu et al., 2020). However, the effect and precise mechanism of QDG on hypertensive vascular dysfunction remain unclear. This study aims to define the role of QDG in the pathogenesis of hypertension and vascular functional and morphological change induced by hypertensive stimuli.

## Methods

### Animals

In this study, 8–10-week-old male C57BL/6 mice (*n* = 25) were purchased from SLAC Laboratory Animal Technology Co., Ltd. (Certificate ID: SCXK 2017-0005, Shanghai, China), and fed in the animal center of Fujian University of Fujian Traditional Chinese Medicine free of water and food, randomly divided into Control, AngII, AngII + QDG-L, AngII + QDG-M and AngII + QDG-H groups (*n* = 5 for each group). Mice in AngII and AngII + QDG- L/-M/-H groups were infused with AngII (500 ng/kg/min), and mice in the Control group were infused with saline for 2 weeks by Osmotic pump. At the same time, mice in AngII + QDG-L/-M/-H groups were given intragastrically three different concentrations of QDG (0.5725, 1.145, or 2.29 g/kg/day, 100 μL for each mice), and in Control and AngII were given intragastrically equal volumes (100 μL) of double distilled water, respectively, for 2 weeks. All the experiments were performed strictly according to the animal ethics in experimental research and approved by the Institutional Animal Care and Use Committee of Fujian University of Traditional Chinses Medicine.

### Qingda Granule Preparation

QDG is composed of the dried herbs: *Gastrodia elata* Blume (Tianma), *Uncaria rhynchophylla (Miz.) Miz. ex Havil.* (Gouteng), *Scutellaria baicalensis* Georgi (Huang Qin), and *Nelumbinis Plumula* Gaertn (Lian Zi Xin) in a ratio of 12: 10: 6: 5. The voucher specimens were also deposited in the Jiangyin Tianjiang Pharmaceutical Co., Ltd. (Jiangsu, China), and the batch number is 1704306 (same batch number as that in the previously published paper by Yu et al. (2020); https://doi.org/10.1016/j.jep.2020.112767). Baicalin has been quantified for controlling the quality of the granule via using Ultra-Performance Liquid Chromatography (UPLC) (Yu et al., 2020). In the UPLC fingerprint profile, the peak of baicalin in QDG was identified by comparing the retention time of the corresponding standard baicalin, revealing baicalin as one of the potential main components of QDG.

For animal experiments, QDG was dissolved in double distilled water to a final concentration (100 μL for each mice) based on the body weight of mice. For cell culture experiments, QDG was dissolved in serum-free media to a concentration of 100 mg/ml. The solution of QDG was freshly prepared just before use.

### Blood Pressure Measurement

The blood pressure of mice was monitored at 0, 1, and 2 weeks after AngII and QDG treatment by a non-invasive tail-vein blood pressure instrument (Kent Scientific, Torrington, CT, United States) according to the instructions. Since various concentrations of QDG treatment exhibit similar attenuation effects on AngII-infused mice, the mice in Ang II + QDG-M group were used for further determination and defined as AngII + QDG.

### Ultrasound Measurement

At the end of treatment, pulse wave velocity (PWV) and thickness of the abdominal aorta in mice from each group were detected by Vevo 2,100 Ultrasound Machine (VisualSonics, Toronto, Ontario, Canada). Briefly, the hair in the abdominal was removed, and the mice were anesthetized with 2% isoflurane and maintained with 1.5% isoflurane. After that, mice were placed in a 37°C pre-heated platform with a heart rate of 450–550 bp. MS400 (30-MHz) probes for mice were placed longitudinally below the sternum and xiphoid process to get the full image of the abdominal aorta in both B mode and M mode. The PWV of each mouse was calculated according to the following formula: length of abdominal aorta/(distal time delay—proximal time delay). Images were taken in both B/M mode, followed by the PWV and thickness of the abdominal aorta via Vevo® LAB software (VisualSonics, Toronto, Ontario, Canada).

### Histological Analysis

Mice were anesthetized by isoflurane and sacrificed. The abdominal aorta was dissected and fixed with 4% paraformaldehyde for 48 h and further embedded in paraffin in a tissue box. The tissues were cut into 4 μm sections and stained with hematoxylin-eosin after rehydration with gradient ethanol.

### RNA Sequencing

The abdominal aorta was dissected and stored in RNAlater (Takara, Beijing, China) at room temperature for 1 h and moved to −20°C for long-time storage. Total RNA was extracted by a mirVana miRNA Isolation Kit (Thermo Fisher Scientific, Grand Island, NY, United States) according to the manufacturer’s protocol, and the concentration and quality were measured by Qubit 3.0 and Agilent 2,100 Bioanalyzer, respectively. RNA samples with a RIN value of seven or above were used for further experiments.

RNA sequencing (RNA-seq) library was built by CapitalBio Technology. In brief, rRNA was removed from total RNA by Ribo-Zero Magnetic Kit according to the instructions. rRNA depleted total RNA was fragmented and generated the poly(A)-tailed mRNA molecules by NEBNext Ploy(A) mRNA Magnetic Isolation Module Kit according to the instructions. Moreover, the final libraries were quantified on Agilent 2,100 Bioanalyzer using KAPA Library Quantification Kit and subjected to paired-end sequencing on an Illumina Hiseq sequencer (Illumina).

The raw data were processed by a bioinformatics pipeline, including the following steps. 1) The sequencing quality was assessed by FastQC (v0.11.5), and low-quality data were filtered by NGSQC (v.2.3.3). 2) Clean reads were aligned to the genome of mice (GCRmm38/mm10 in UCSC) by HISAT2 (v2.1.0) with default paraments. 3) Reconstruction of genes and transcripts based on the results of reads comparison and quantification of genes were performed using StringTie (v1.3.3b). 4) Bio MAS (molecule annotation system v3.0) was used for correlation analysis between samples and functional annotation of genes. 5) The differentially expressed transcripts (DETs, with the condition of |fold change| ≥ 2 and *p* < 0.05) between groups were analyzed using DESeq (v1.28.0). 6) The selected genes were further analyzed in the context based on the information obtained from the database of gene ontology (GO) and Kyoto Encyclopedia of Genes and Genomes (KEGG).

### Immunohistochemical Staining

Immunohistochemical (IHC) staining was performed with 4 μm sections of the abdominal aorta. After being rehydrated with gradient ethanol, the tissues were treated with 0.01 M citrate antigen retrieval solution in a pressure cooker with high temperature for 15 min and cooled down to room temperature and then were incubated with primary antibody for phospho-ERK (p-ERK; 1:200) in a wet box at 4°C overnight. After being washed with PBS for three times, the tissues were incubated with a secondary antibody for 1 h and DAB staining according to instructions. The images were taken randomly in six independent areas of each sample using Leica DM4000B intelligent automated optical Microscope (Leica, Wetzlar, Germany) and analyzed by true color multi-functional cell image analysis system (Image-Pro Plus, Media Cybernetics, Rockville, MD, United States). IHC scores were calculated using the following formula: positive cells × staining intensity. There are 5 grades for positive cells: 0, <10% cells were positive; 1, 10–25% cells were positive; 2, 25–50% cells were positive; 3, 50–75% cells were positive; 4, >75% cells were positive. There are 3 grades for staining intensity under the optical microscope: 1, weak (light yellow); 2, intermediate (light brown); 3, strong (dark brown).

### Vascular Smooth Muscle Cells Isolation and Identification

Primary vascular smooth muscle cells (VSMCs) were obtained as previously described (Mu et al., 2018). Briefly, rats were anesthetized by isoflurane and the abdominal aorta was dissected immediately and washed with ice 1.5 mM of CaCl_2_-HEPES-buffered salt solution (HBSS) to remove the blood. Then, the aorta was cut longitudinally and swiped slightly by a cotton swab to remove the endothelial cells. After being placed in 1.5 mM CaCl_2_ HBSS for 30 min at 4°C and Ca^2+^-free HBSS for 20 min at room temperature for 20 min, the aorta was digested in the 0.5 ml pre-prepared mix enzyme-containing collagenase Ⅰ (3.2 mg), papain (0.3 mg), and BSA (2 mg) for 20–30 min at 37°C. After removing the extra enzyme, the aorta was washed with Ca^2+^-free HBSS and placed in the T25 flask with 4 ml DMEM supplemented with 10% FBS and 1% penicillin/streptomycin. The VSMCs were dispersed by piping up and down 50–60 times gently and cultured at an incubator with 5% CO_2_.

### CCK8 Assay

VSMCs in suspension (100 μL) were seeded into 96-well plates at a density of 2 × 10^4^ cells/ml. Then, VSMCs were treated with different concentrations of QDG (12.5, 25, and 50 μg/ml) for 48 h. At the end of treatment, 10 μL of CCK-8 solution was added to each well, and the plate was incubated for another 2 h at 37°C in air containing 5% CO_2_. Absorbance was measured at 450 nm using a Microplate Reader (Multiskan FC, Thermo Fisher Scientific, Grand Island, NY, United States).

### Measurement of (Ca^2+^)i

Determinations of (Ca^2+^)i were performed as previously described (Mu et al., 2018). Briefly, cultured VCMCs were pre-treated with QDG (50 μg/ml) or PBS for 48 h and then incubated with 5 μM of the Ca^2+^-sensitive fluorescent dye Fluo-4 for 30 min at room temperature in the dark. The extracellular dye was washed with Ca^2+^-free HBSS 3 times, and cells were visualized using a PerkinElmer Ultraview spinning disk confocal microscope (PerkinElmer Inc., Waltham, United States). After recording the basal fluorescence of cells in 90 s (F_0_), 100 nM of AngII was added, and the change of fluorescence was recorded for another 15 min (F_1_). All the images were processed using Velocity software (PerkinElmer Inc., Waltham, United States), and the change of fluorescence was shown as F_1_-F_0_.

### Western Blotting

Western blotting was performed as previously described (Shen et al., 2018). Briefly, cultured VSMCs cells were seeded in a 6-well plate at the concentration of 1 × 10^5^ cells/ml and treated with various concentrations of QDG (12.5, 25, and 50 μg/ml) or PBS for 48 h and then treated with 100 nM of AngII for 8 min. After the treatment, cells were lysed for western blotting by IP lysate buffer with 1 mM phenylmethylsulfonyl fluoride and protease inhibitors on ice for 30 min. Total protein concentration was detected by BCA assay and 40 μg of proteins of each sample was separated by SDS-PAGE and transferred onto a PVDF membrane by a wet transfer system. Then, the membranes were blocked by 5% non-fat skimmed milk at room temperature for 2 h and incubated with primary antibodies (anti-p-ERK 1:1,000, anti-ERK 1:1,000, anti-GAPDH 1:5000) at 4°C overnight. After being washed with TBST three times, the membranes were incubated with secondary antibody at room temperature for 1 h and detected by an ECL kit. Then, the membranes were placed in Restore Western Blot Stripping Buffer and incubated for 30 min at 37°C. After being washed with TBST, the membranes were blocked, incubated with primary antibodies (anti-ERK 1:1,000) at 4°C overnight, and then incubated with secondary antibody at room temperature for 1 h. Finally, an ECL kit was used to detect protein expression. The intensity was analyzed in Image J, and GAPDH was set as the internal control.

### Statistical Analysis

Statistical analysis of this study was performed using SPSS 26.0 (IBM). All the data were shown as mean ± SD and detected the normality by Shapiro–Wilk test. One-way ANOVA and Kruskal–Wallis test were used for detecting the statistical significance for normal distribution and nonparametric data, respectively. *p < 0.05* was recognized as statistically significant.

## Results

### Qingda Granule Lowers Blood Pressure of AngII-Induced Hypertensive Mice

**FIGURE 1:**
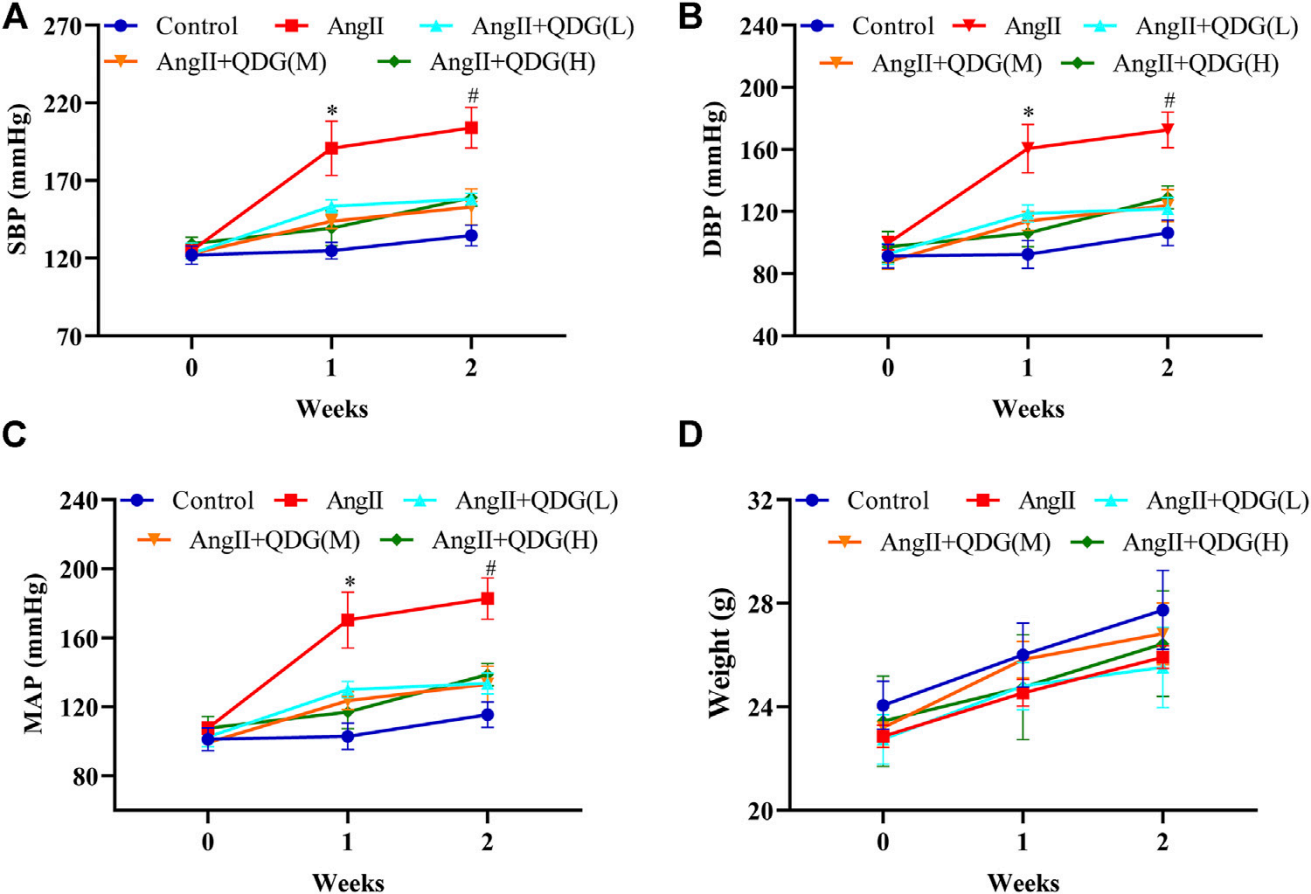
Qingda granule (QDG) lowers the blood pressure of angiotensin- (AngII-) induced hypertensive mice. Systolic blood pressure **(A)**, diastolic blood pressure **(B)**, mean arterial pressure **(C)**, and body weight **(D)** of mice from each group. Data are presented as mean ± SD; *n* = 5 for each group; *p < 0.05 vs. Control group, #*p* < 0.05 vs. AngII group.

To investigate the functional role of QDG treatment on antihypertension, we first detected the effect of QDG (0.5725, 1.145, or 2.29 g/kg/day) treatment on blood pressure of AngII-induced hypertensive mice. The blood pressure, including systolic blood pressure (SBP), diastolic blood pressure (DBP), and mean arterial pressure (MAP), was increased in AngII-infused mice, which was reversed by different concentrations of QDG treatment (Figures 1A–C), while no difference was found between different concentrations of QDG treatment. Moreover, there was no significant difference in body weight among all groups (Figure 1D). These results suggest that QDG treatment lowers the blood pressure of AngII-induced hypertensive mice and didn’t affect body weight.

### Qingda Granule Attenuates Vascular Functional and Pathological Change of AngII-Induced Hypertensive Mice

**FIGURE 2:**
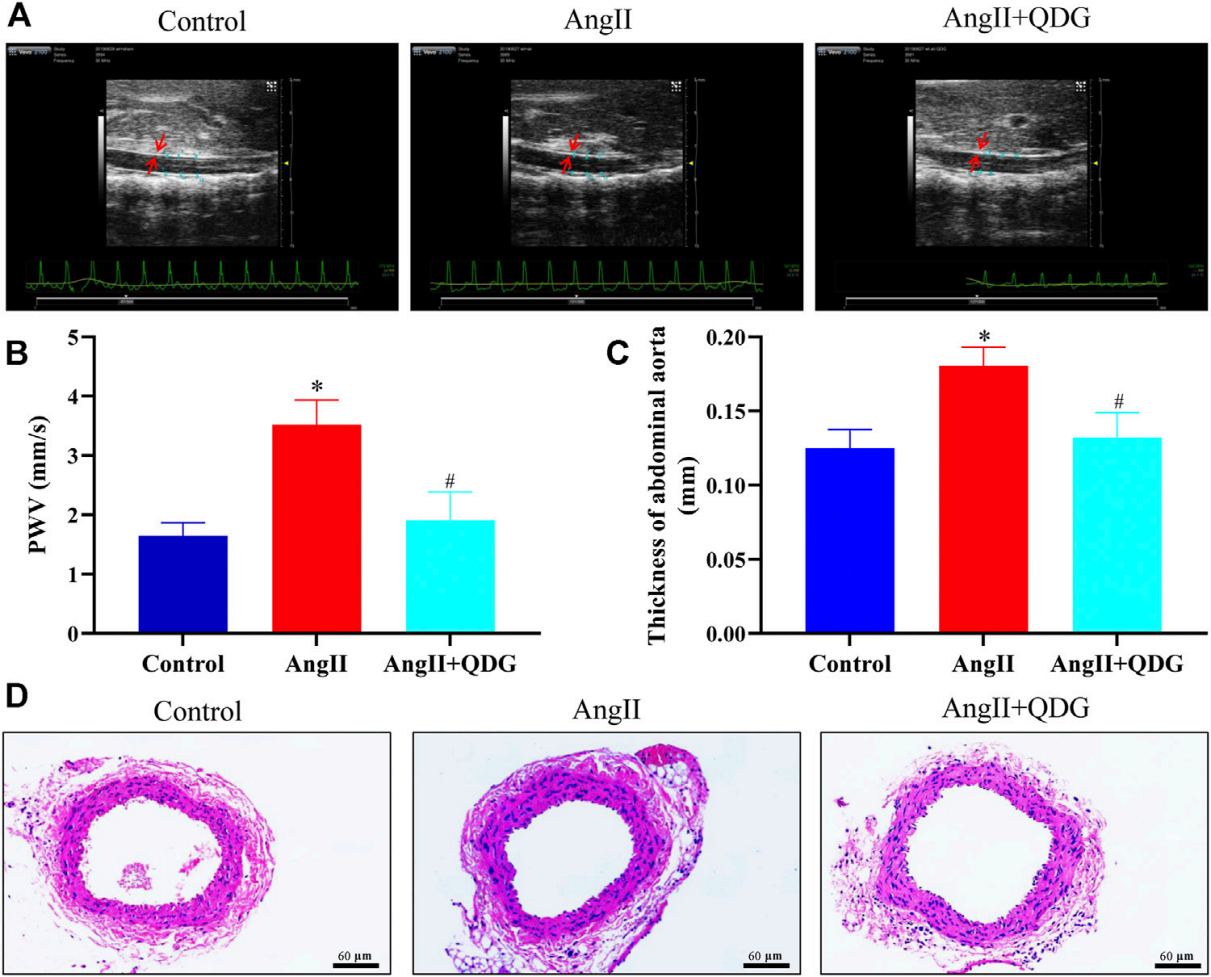
Qingda granule (QDG) attenuates vascular functional and pathological change of angiotensin- (AngII-) induced hypertensive mice. **(A)** Representative images of ultrasound in each group. PWV **(B)** and wall thickness **(C)** of the abdominal aorta were analyzed. **(D)** H&E staining of abdominal aorta (scale bar = 60 um). Data are presented as mean ± SD; *n* = 5 for each group; *p < 0.05 vs. Control group, #*p* < 0.05 vs. AngII group.

To assess the effect of QDG treatment on vascular function of hypertensive mice, we examined the PWV of the abdominal aorta in each mouse by animal ultrasound. The PWV of the abdominal aorta was increased after 2 weeks of AngII infusion and attenuated by QDG treatment (Figures 2A,B). Furthermore, the thickness of the abdominal aorta was increased by AngII infusion and decreased by QDG treatment (Figure 2C); this result was further confirmed by HE staining (Figure 2D). These results suggest that QDG treatment attenuate vascular functional and pathological change in AngII-induced hypertensive mice.

### Genome-Wide Gene Expression Profiling

**FIGURE 3:**
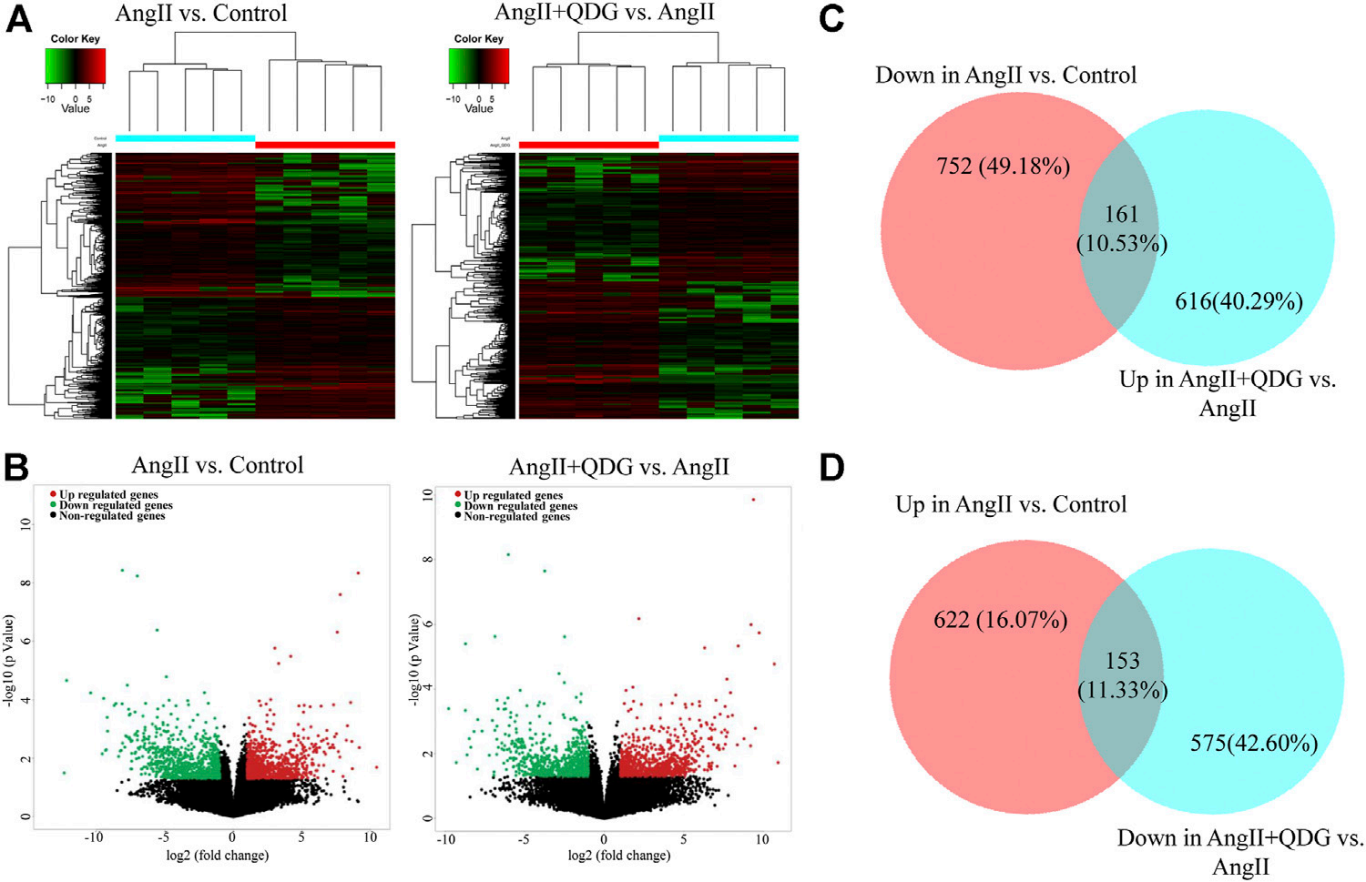
Qingda granule (QDG) induces change of gene expression profiles. After 2 weeks of treatment of AngII and QDG, the abdominal aorta was collected and detected the DEGs (|fold change| ≥ 2 and *p* < 0.05) by RNA sequencing of AngII compared to Control **(A)** and AngII + QDG compared to AngII **(B)**. **(C,D)** The overlapped area represented 314 of 1688 altered genes by AngII stimulation, which were attenuated by QDG treatment.

To further determine the underlying molecular mechanism of QDG treatment, we performed RNA-seq to detect the gene expression profiles of abdominal aorta in each group, and cluster maps (Figure 3A) and volcano maps (Figure 3B) were drawn according to DETs (GSE165601; https://www.ncbi.nlm.nih.gov/geo/query/acc.cgi?acc=GSE165601). Total 775 transcripts were upregulated and 913 transcripts were downregulated after AngII infusion, whereas 777 transcripts were upregulated and 728 transcripts were downregulated after QDG treatment compared to AngII group. Integrative analysis indicated that upregulated 161 transcripts and downregulated 153 transcripts induced by AngII were reversed by QDG treatment (Figures 3C,D).

### Gene Ontology Analysis and Kyoto Encyclopedia of Genes and Genomes Pathway Annotation

**FIGURE 4:**
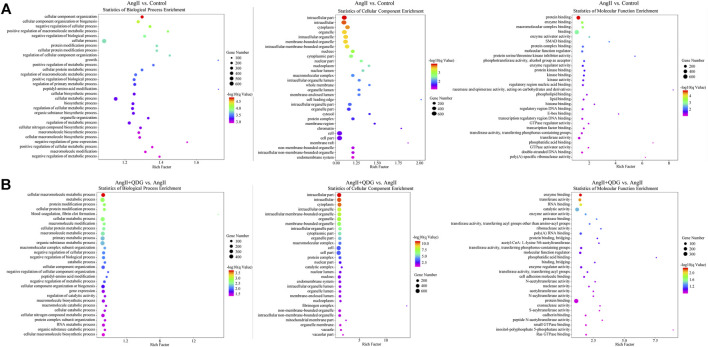
Gene Ontology (GO) enrichment analysis. The top 30 enrichment items were taken to analyze the related biological processes, cellular composition, and molecular function of **(A)** AngII vs. Control and **(B)** AngII + Qingda granule (QDG) vs. AngII.

GO analysis can help identify characteristic biological attributes from the aspects of biological processes, cellular composition, and molecular function for DETs. The top 30 enriched GO terms are shown in Figures 4A,B. In terms of biological processes, they mainly exhibited enrichment for metabolism, energy pathway, and regulation of cell growth. Cell component analysis revealed mRNA was mainly distributed in the cytoplasm, organelle, and nucleus. The molecular function was enriched in enzyme binding, transferase activity, and molecular function regulator.

**FIGURE 5:**
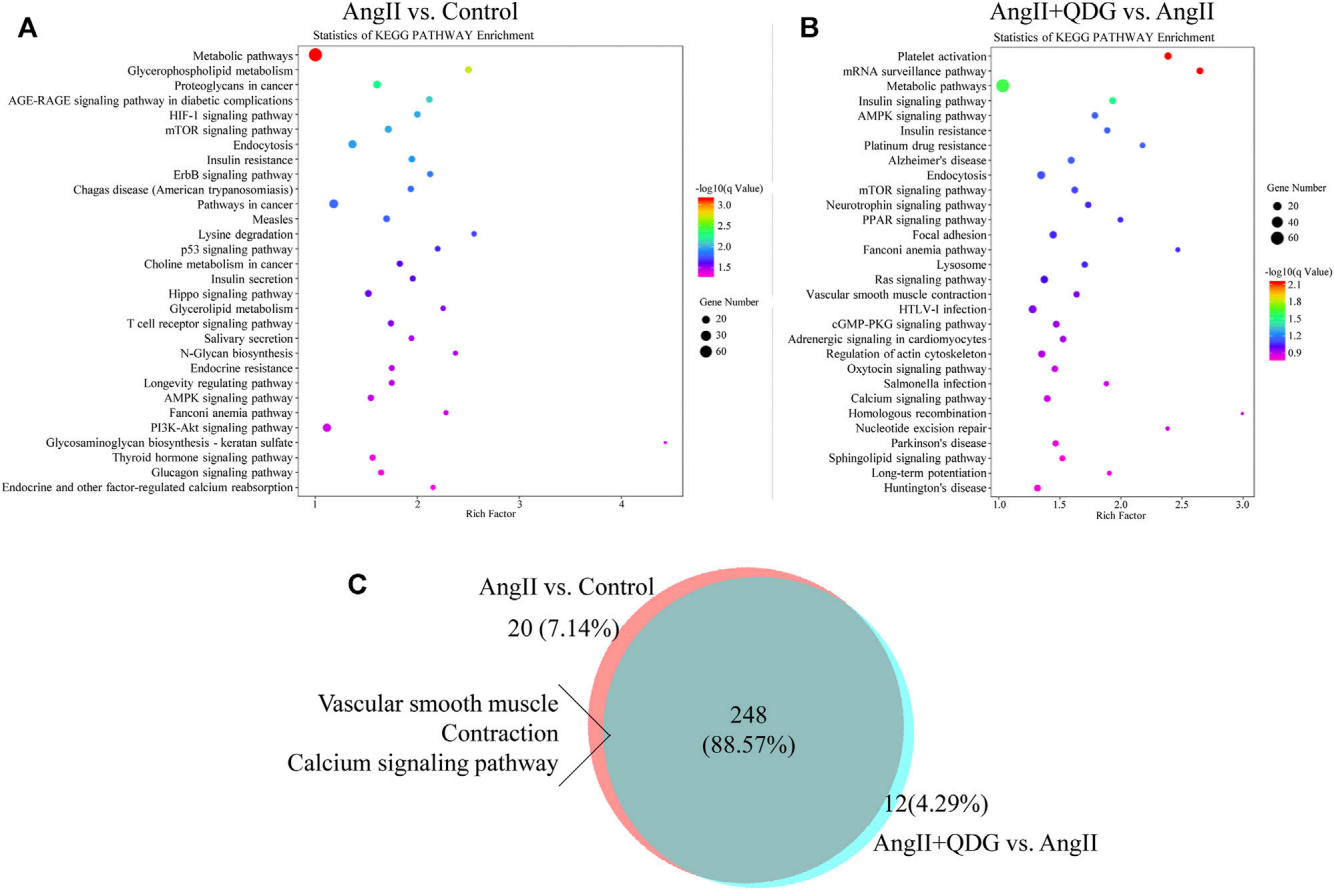
Kyoto Encyclopedia of Genes and Genomes (KEGG) enrichment analysis. The top 30 enrichment KEGG signaling pathway analysis was performed to identify related signaling pathways of DETs after AngII **(A)** and QDG **(B)** treatment. **(C)** 248 altered pathways by AngII treatment were attenuated by QDG treatment.

Furthermore, we performed KEGG pathway enrichment analysis to explore further the pathological mechanism of QDG in the treatment of AngII-induced hypertension and identify the related pathways to the functions of AngII vs. Control and AngII + QDG vs. AngII. The top 30 most significantly enriched signaling pathways are shown in Figures 5A,B. Among all of the enriched pathways between AngII vs. Control and AngII + QDG vs. AngII, there were 248 overlapping pathways (Figure 5C). Among the overlapped pathways, calcium signaling pathway and ERK related vascular contraction were enriched, suggesting QDG treatments may exert its functional role on hypertension through calcium and related ERK pathways.

### Qingda Granule Inhibits Ca^2+^/ERK Signaling Pathway Activated by AngII

**FIGURE 6:**
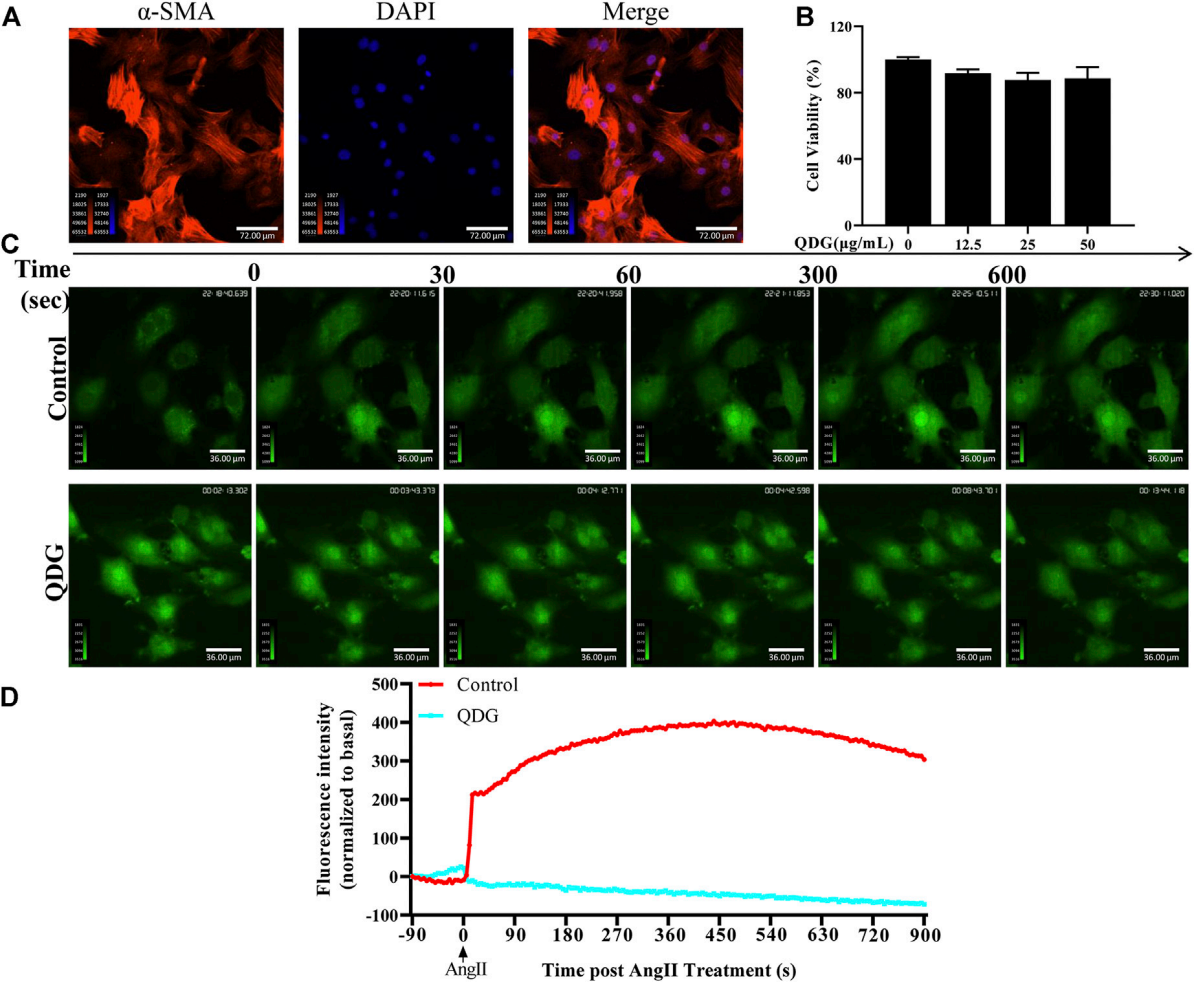
Qingda granule (QDG) inhibits Ca^2+^ release induced by angiotensin (AngII). **(A)** Immunofluorescence staining of *α*-SMA was used to confirm that primary cultures contained rat vascular smooth muscle cells (VSMCs) under microscopy (200×). Images are representative of those obtained for three independent cultures. **(B)** CCK8 was performed to determine the cell viability of VSMCs after 12.5, 25, or 50 μg/ml of QDG treatment. The cell viability of untreated cells was set as 100%. **(C)** VSMCs were incubated with PBS or QDG (50 μg/ml) for 48 h then treated with 100 nM AngII to record the real-time Ca^2+^ concentration by confocal microscope. **(D)** Intracellular Ca^2+^ concentration was normalized to basal before AngII treatment.

Based on the RNA-seq results, we confirmed the regulatory role of QDG in AngII-induced Ca^2+^/ERK signaling pathway *in vitro*. Isolated primary VSMCs of the abdominal aorta were identified by detecting the expression of α-SMA, which showed that over 95% of the cells were positive for this antibody and identified as VSMCs (Figure 6A). Moreover, CCK8 analysis revealed that 12.5, 25, and 50 μg/ml of QDG treatment exhibited without or minor effect on cell viability of VSMCs, and therefore, 50 μg/ml of QDG was selected for determination of Ca^2+^ release. As shown in Figures 6C,D, intracellular Ca^2+^ was increased after AngII stimulation in VSMCs, while it was attenuated with the treatment of QDG (50 μg/ml), suggesting that QDG treatment inhibits the AngII-induced intracellular Ca^2+^ release.

**FIGURE 7:**
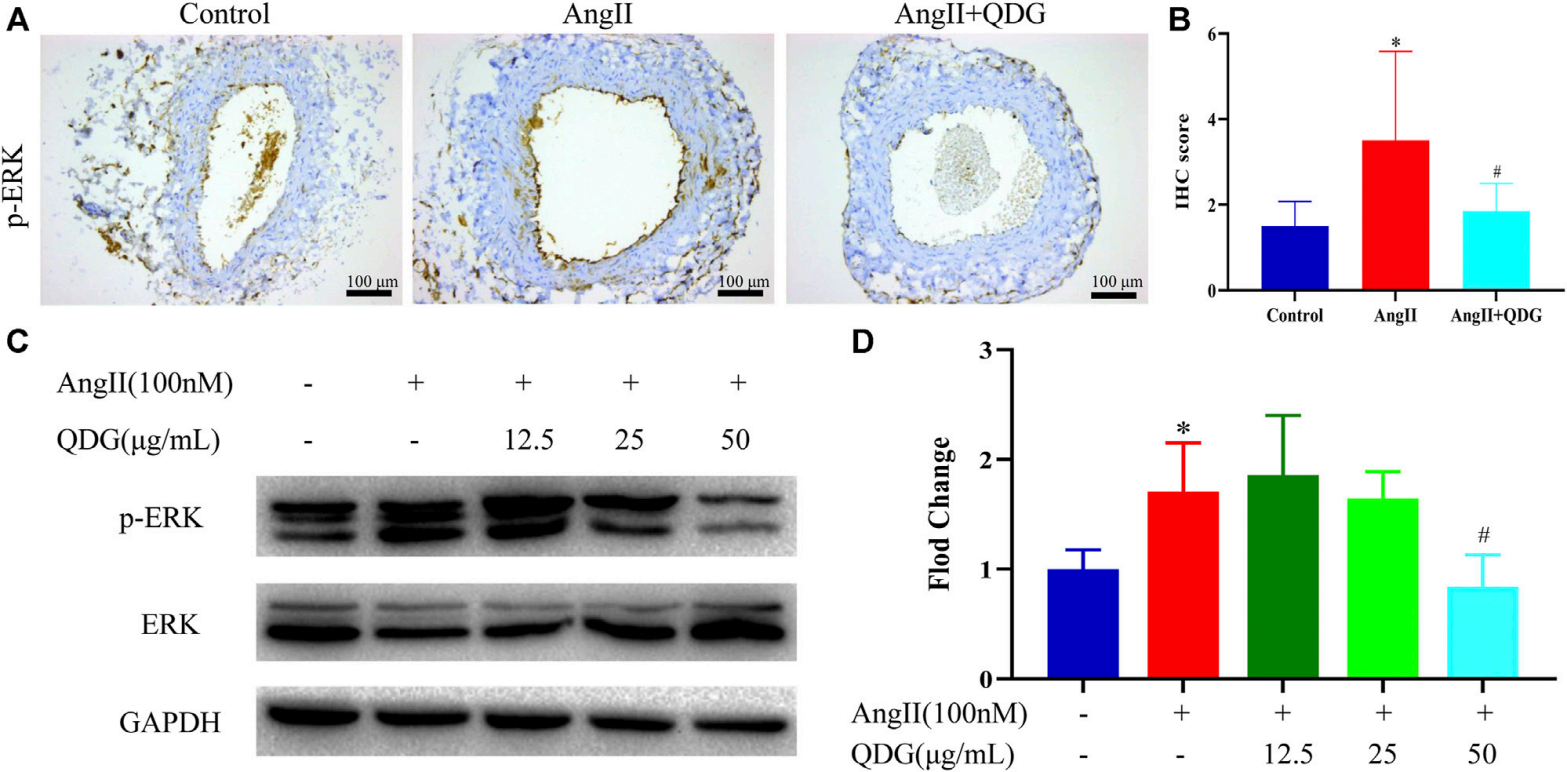
Qingda granule (QDG) inhibits ERK signaling pathways induced by angiotensin (AngII). **(A)** Immunohistochemical staining of phospho-ERK (p-ERK) in the abdominal aorta (scale bar = 100 um) and its intensity **(B)** was analyzed. **(C)** Western blotting of p-ERK and ERK in primary VSMCs and intensity ratio of p-ERK and ERK **(D)** was analyzed. Data are presented as mean ± SD; *n* = 5 for *in vivo* study and *n* = 3 for *in vitro* study; *p < 0.05 vs. Control group, #*p* < 0.05 vs. AngII group.

We detected the p-ERK expression both *in vivo* and *in vitro*. As shown in Figures 7A,B, p-ERK expression in the abdominal aorta of mice was upregulated after 2-week AngII infusion and blocked by QDG treatment (Figures 7A,B). Consistent with the *in vivo* study, p-ERK expression was increased in VMSCs after AngII treatment for 48 h and blocked by QDG treatment without disturbing the ERK expression (Figures 7C,D).

## Discussion

Despite improved awareness and therapies of hypertension, the number of hypertensive patients with controlled blood pressure remains low (Wang et al., 2018). Therefore, developing new effective pharmacological therapies is an urgent issue in this area. This study provides novel evidence that QDG treatment lowers the blood pressure of hypertensive mice and rescues the vascular dysfunction caused by hypertension. Mechanically, QDG treatment reverses 314 of 1688 altered genes and 248 of 268 modified signaling pathways by AngII stimulation, among which Ca^2+^ and related ERK signaling pathways may be one of the ways of QDG to exert its anti-hypertensive effect.

QDG has been proven to lower blood pressure and restore the cardiac function of hypertensive rats (Wu et al., 2020). In this study, using the AngII-induced mice model, we found that various concentrations of QDG treatment lowered the blood pressure, including SBP, DBP, and MAP of hypertensive mice, which provided novel evidence for the anti-hypertensive effect of QDG. Overload pressure causes the vascular functional and structural changes of the aorta, which in turn promotes the progression of hypertension (Cohen 2007). PWV was used to clinically evaluate the vascular function of hypertension patients (Aksenova et al., 2018). Our results indicated AngII induced the increased PWV of mice which can be decreased by QDG treatment, suggesting QDG treatment can partly rescue hypertensive vascular dysfunction. At the same time, both ultrasound and H&E staining demonstrated that QDG treatment can decrease the thickness of the abdominal aorta of hypertensive mice. Taking all these results together, QDG treatment lowers the blood pressure and attenuates vascular functional and structural change induced by hypertension.

The development of the RNA-seq technique provides a useful means to explore the underlying molecular mechanism of disease (Pareek et al., 2011). Therefore, RNA-seq was performed to investigate the DETs and related signaling pathways after AngII and QDG treatment, which may contribute to the anti-hypertensive effect of QDG. We found QDG treatment reversed multiple genes and signaling pathways altered by AngII, including PPAR, mTOR, and RAS signaling pathway, among which calcium signaling pathway and ERK-mediated vascular smooth muscle contraction were associated with vascular dysfunction (Touyz, 2005); that is, AngII mediates Ca^2+^ release from SR and subsequently activates ERK signaling pathway (Mehta and Griendling, 2007). Therefore, we explored whether QDG treatment exerts its functional role on hypertension and vascular dysfunction through this pathway.

Consistent with the results of RNA-seq, QDG treatment significantly decreased the intercellular Ca^2+^ concentration induced by AngII in isolated VSMCs. Furthermore, QDG treatment also decreased the protein level of p-ERK induced by AngII both *in vivo* and in *vitro*. These results suggest that QDG treatment alleviates Ca^2+^ and related ERK signaling pathway, which may partly explain how QDG lowers blood pressure and attenuates hypertensive vascular dysfunction. However, the inhibitors of Ca^2+^ and ERK pathway should be further used to confirm the anti-hypertensive role of QDG in future studies definitively.

## Conclusion

In summary, this study demonstrates that QDG treatment lowers blood pressure and attenuates hypertension-induced vascular functional and structural changes of hypertensive mice. The possible underlying molecular mechanism is that QDG treatment inhibits AngII-induced Ca^2+^ and related ERK signaling pathway, which provides novel evidence of QDG for anti-hypertension.

## Data Availability

The datasets presented in this study can be found in online repositories. The names of the repository/repositories and accession number(s) can be found in the article/Supplementary Material.

## Abbreviations

AngII, angiotensin II; AT1R, AngII type I receptor; DAPI, 4’,6-diamidino-2-phenylindole; DBP, diastolic blood pressure; DETs, differentially expressed transcripts; HBSS, HEPES-buffered salt solution; GO, gene ontology; KEGG, Kyoto Encyclopedia of Genes and Genomes; MAP, mean arterial pressure; p-ERK, phospho-ERK; PWV, pulse wave velocity; QDG, Qingda granule; QXJYD, Qingxuan Jiangya Decoction; RAAS, renin-angiotensin-aldosterone system; RNA-seq, RNA sequencing; SBP, systolic blood pressure; SHRs, spontaneously hypertensive rats; TCM, traditional Chinese medicine; UPLC, Ultra-Performance Liquid Chromatography; VSMCs, vascular smooth muscle cells.
